# Regulatory B cells, the key regulator to induce immune tolerance in organ transplantation

**DOI:** 10.3389/fimmu.2025.1561171

**Published:** 2025-04-08

**Authors:** Jinfeng Liao, Yixin Yang, Jisong Li, Zheng Liu, Siyuan Song, Yu Zeng, Yi Wang

**Affiliations:** ^1^ Department of Dermatology, Sichuan Provincial People’s Hospital, University of Electronic Science and Technology of China, Chengdu, Sichuan, China; ^2^ Department of Clinical Medicine, The First Clinical Medical College of Norman Bethune University of Medical Sciences, Jilin, China; ^3^ Department of Gastrointestinal Surgery, Sichuan Provincial People’s Hospital, University of Electronic Science and Technology of China, Chengdu, Sichuan, China; ^4^ Department of Pathology, MD Anderson Cancer Center, Houston, TX, United States; ^5^ Department of Neuroscience, Baylor College of Medicine, Houston, TX, United States; ^6^ Department of Hyperbaric Oxygen, Sichuan Provincial People’s Hospital, University of Electronic Science and Technology of China, Chengdu, Sichuan, China; ^7^ Department of Critical Care Medicine, Sichuan Provincial People’s Hospital, University of Electronic Science and Technology of China, Chengdu, Sichuan, China; ^8^ Translational Clinical Immunology Key Laboratory of Sichuan Province, Sichuan Academy of Medical Sciences and Sichuan Provincial People’s Hospital, Chengdu, China

**Keywords:** regulatory B cells (Breg), interleukin-10 (IL-10), solid organ transplantation, chemokines, B cell differentiation, immune tolerance

## Abstract

In solid organ transplantation, especially renal transplantation, for the induction of immune tolerance, accumulating evidence has revealed that Regulatory B cells (Breg) play a crucial role in stimulating immune tolerance, alleviating immune responses, and improving graft survival. We describe the heterogeneous nature of Bregs, focusing on their defining surface markers and regulatory functions. Meanwhile, the major cytokine secretion function and the correlation between Breg and Treg or other immune checkpoints to balance the immune responses are addressed. Furthermore, we summarized the intrinsic and extrinsic pathways or costimulatory stimuli for the differentiation from naïve B cells. More importantly, we summarized the progression of the immune tolerance induction role of Breg in solid organ (kidney, liver, heart, lung, and islet) transplantation. This is an up-to-date review from the origin of Breg to the function of Breg in solid organ transplantation and how it induces immune tolerance in both murine models and human solid organ transplantation.

## 1 Introduction

In organ transplantation, the recipient’s immune system has three major responses against the grafts (1), which are acute rejection, chronic rejection, and graft-versus-host disease (GVHD) (2). These immune responses are primarily initiated by T cell-mediated rejection, where donor antigens (particularly major histocompatibility complex (MHC) molecules) on the graft are recognized by the recipient’s immune cells. For acute rejection, T cells and antibodies recognize and attack the graft within days or weeks after transplantation (3). Whereas chronic rejection is a prolonged and ongoing immune response that leads to gradual loss of graft function over time (4). These two immune responses are mostly in solid organ transplantation, and the immune response is the rejection of grafts, called host-versus-graft disease (HVGD) (5). However, for bone marrow and hematopoietic stem cell transplantation, there will be GVHD, a complication where donor immune cells attack the recipient’s tissue (6). Therefore, to prevent these immune responses, immune tolerance to the graft has been investigated and established for years, whether in allogenic or xenogeneic transplantation.

For the induction of immune tolerance, accumulating evidence has revealed that Regulatory B cells (Breg) play a crucial role in stimulating immune tolerance, alleviating immune responses, and improving graft survival (7, 8). Bregs are a subset of B cells identified as having an immunosuppressive function, modulating the immune system to prevent excessive inflammation and autoimmune diseases (9, 10). Contrary to the role of B cells, which have traditionally been associated with antibody production and antigen presentation, Breg has been documented to contribute to immune homeostasis by regulating T cell responses (11) and anti-inflammatory cytokine production (12). Therefore, in this review, we comprehensively summarize the specific role of Breg in organ transplantation, including solid organ transplantation and hematopoietic transplantation.

## 2 Characteristics of Breg

Breg are a subset of B cells, which are not a uniform population, but rather a diverse group of cells (13). To date, there is no single marker universally accepted to identify all Breg, but several characteristics can define this subset of cells. The first one is CD19^+^CD25^+^CD1d^+^ cells (14–16). These cell surface markers are often associated with regulatory B cells, even though they are not specific to Breg only. The second one is CD24^hi^CD38^hi^ cells (17–20). The third one is IL10^+^ cells (21, 22). There is a discrepancy in CD39^hi^ (23, 24) and CD39^-^ (25) cells are Breg, therefore, CD39 might not be a canonical marker for Breg. The fourth one is IL-35 secreting B cells. This subset of B cells has been shown to suppress autoimmune diseases (26, 27), including autoimmune diabetes (28), systemic lupus erythematosus (29), ankylosing spondylitis (30), thyroid associated opthalmopathy (31) and also CNS (central nervous system) autoimmune disease (multiple sclerosis and uveitis infection) (32) as chronic hepatitis B (33). Further, IL-35-producing Breg could suppress inflammation and alveolar bone resorption in ligature-induced periodontitis (11). There is another subset of B cells that produce granzyme B, identified with CD307b^hi^, CD258^hi^CD72^hi^, and CD21^lo^PD-1^hi^ B cell subpopulations (34). There are other subtypes of Breg, detailed addressed in **Table 1**.

**Table 1:** Breg characteristics and their specific functions.

Subsets of Bregs	Transplantation or other disease	Function	References
CD19+CD25+CD1d+ cells	Renal transplantation	positively correlated with better graft function and longer and higher Treg level	(14)
CD19+CD24hiCD38hi cells	Renal transplantation	long-term graft survival of renal transplantation	(126)
	Renal transplantation	long-lasting graft survival of renal transplantation with drug-free	(128)
	Renal transplantation	longer survival with belatacep after renal transplantation	(131)
	Lung transplantation	Long-term lung grafts survival	(20)
CD19+CD24hiCD27+ Breg	Liver transplantation	predict the occurrence of acute allograft rejection in liver transplantation	(134)
CD19+CD5+CD1dhi Breg	Heart transplantation	Protective role in heart transplantation	(136)
	Islet transplantation	Responsible for the early stage of transplantation tolerance induction	(16)
CD39hi	Induce cytokine secretion	Induce IL-10 secretion	(23, 24)
CD39-	Breast cancer	Limited Th proliferation, type-1 cytokine production, and Teff survival. Stimulate Treg	(25)
B220+/Tim1+ Breg	Islet transplantation	Induce islet transplantation tolerance	(121)
CD19+TIM-1+Breg	Islet transplantation	Critical in the whole process of tolerance induction and maintenance	(16)
IL10+ B cells	Renal transplantation	Mouse model of renal transplantation, modulating T cell responses	(73, 124)
	Renal transplantation	Alleviate acute rejection of renal transplantation	(125)
	Renal transplantation	Alleviate renal injury after transplantation	(95)
	Renal transplantation	Prolong graft survival by decreasing CD3+ T cell proliferation	(127)
	Renal transplantation	Non-immunosuppressant for at least 1 year after renal transplantation	(129)
TGF-β-producing B cells	Renal transplantation	Tolerant drug-free patients with drug free	(130)
	Islet transplantation	Establish islet transplantation tolerance	(73)
IL-35-producing B cells	Autoimmune disease	Autoimmune diabetes	(28)
	Autoimmune disease	Systemic lupus erythematosus	(29)
	Autoimmune disease	Ankylosing spondylitis	(30)
	Autoimmune disease	Thyroid associated opthalmopathy	(31)
	Autoimmune disease	Multiple sclerosis and uveitis infection	(32)
	Autoimmune disease	Chronic hepatitis B	(33)
	Autoimmune disease	Periodontitis	(11)
granzyme B-producing B cells	Renal transplantation	Maintain allo-specific tolerance	(132)

The main immune regulatory function of Breg is realized by producing anti-inflammatory cytokines, interleukin-10 (IL-10) (35, 36), IL-35 (26, 27), IDO (37) and granzyme B (38, 39). As an anti-inflammatory cytokine, IL-10 is known for its immune-regulatory properties, such as inhibiting the activation of T cells (40), dendritic cells (41), and macrophages (42), and suppressing the production of pro-inflammatory cytokines (43). IL-10 can suppress inflammatory responses and the activation of immune cells, thereby regulating the inflammatory immune response. It primarily works by inhibiting the migration, infiltration, proliferation, and activation of inflammatory cells (44), and by suppressing the production of pro-inflammatory factors by Th1 cells (45, 46), thus inhibiting the cellular immune response (22). In addition, IL-10 can inhibit activated monocytes from secreting interleukin-1 (IL-1) and interleukin-6 (IL-6) (47), suppress the release of TNF-α by macrophages (48), and inhibit the activation of mast cells and the secretion of their cytokines (49, 50), thereby participating in the regulation of allergic reactions (40, 51). IL-10 also has an activating effect on B cells and can promote antibody production (52).

For the IL-35-producing Breg, they could be induced by IL-12p35 (53). Meanwhile, the production of IL-35 by Breg is facilitated through the binding of the BATF-IRF-4-IRF-8 complex to the promoter elements of the il12a and ebi3 genes (54). In the lung tissue of OVA-induced asthmatic mice, IL-35 enhances the presence of Breg that co-express IL-35 and IL-10, as well as conventional LAG3^+^ regulatory T cells (55).

Breg also produces granzyme B. In liver transplant recipients with acute rejection, CD19^+^ granzyme B-producing Breg serves as a feedback loop to modulate the activation of CD4+CD25- T cells (56). Due to the significance of granzyme B-producing Breg, S. Brouard laboratory generated a novel protocol to expand this subtype of Breg (39). The dysfunction of granzyme B-producing Breg is associated with more severe rheumatoid arthritis (38). Meanwhile, human granzyme B-producing Breg could inhibit the proliferation of effector CD4^+^CD25^-^ T effector cells (57).

## 3 The differentiation of Breg from naïve B cells

The differentiation of Breg from naïve B cells is via both intrinsic and extrinsic signals. B cells can be activated by the recognition of antigens through the B cell receptor (BCR) and also co-stimulatory signals (58). During this process, the BCR recognizes a specific antigen, typically in the form of proteins or polysaccharides on the surface of pathogens, dead cells, or other stimuli (59). Then, this antigen binds to the BCR to initiate intracellular signaling through the spleen tyrosine kinase (Syk) pathway (60) and subsequently activates downstream PLCγ2, PI3K, MAPK pathways (61, 62). With the activation of these signaling pathways, B cells are activated, proliferate, and survive. Beyond antigen recognition, Breg require co-stimulatory signals for full activation (23, 63). CD40 signaling is a crucial co-stimulatory pathway that activates B cells (64). CD40 ligation by CD40L (present on T cell surface) triggers several downstream signaling events that influence the differentiation program of the B cell, including NF-κB activation, which is crucial for B cell survival and activation (65–67). Several transcription factors are involved in guiding naïve B cells to adopt a regulatory phenotype, and they form part of the intrinsic genetic pathway for Breg differentiation. Several transcription factors contribute to the development of IL-10-producing Breg, including Bach2 (68), BRD4 (69), the Nuclear Factor Kappa-B (NF-κB) signaling pathway (70), Interferon Regulatory Factor (IRF4) (54), STAT3 and c-MAF (71), Foxp3 (72), Transforming Growth Factor-beta (TGF-β) signaling (73, 74), IL-21 (75, 76) (a cytokine produced by T helper type 17 (Th17) and follicular helper T (Tfh) cells), and B Lymphocyte-Induced Maturation Protein 1 (BLIMP-1) (77). Furthermore, toll-like receptors (TLRs) are crucial for the induction of IL-10-producing Breg (78). By antigen-presenting cells (APCs), signals from TLRs are essential for IL-10 production. Autophagosomes released by tumors stimulate the formation of IL-10-producing Breg, which in turn suppress T lymphocyte activity through the TLR2-MyD88-NF-κB signaling pathway (79). For the generation of granzyme B-producing Breg, it could be generated by B-chronic lymphocytic leukemia (B-CLL) cells treated with interleukin-21 (IL-21) (80). CD4^+^ T cells can produce IL-21 and rapidly induce granzyme B-producing Breg in co-cultured B cells in an IL-21 receptor-dependent manner (81, 82). Lymphotoxin alpha, a new and potent Breg ligand, has also been reported to increase granzyme B expression in Breg (57).

The extrinsic pathway refers to the external signals from the microenvironment, such as cytokines, cellular interactions, and immune stimuli, that influence the differentiation of naïve B cells into Breg. These extrinsic signals include immune cells (T cells, dendritic cells, and macrophages) (83–86), cytokines produced during inflammation or tolerance induction, and tissue-specific microenvironments (23, 87). For the cytokine-driven Breg differentiation it includes IL-10, TGF-β, and IL-21, along with interactions with T cells, dendritic cells, and other immune cells, providing additional stimuli for the differentiation of Breg. Moreover, Indoleamine 2, 3-dioxygenase (IDO) could be generated by Breg (37), and in turn, it could induce Breg infiltration in lung cancer (88). Mesenchymal stromal cells alleviate multiple sclerosis by increasing the suppressive proportion of CD5^+^ IL-10^+^ Breg in an IDO-dependent manner (55).

## 4 The function of the Breg

The primary function of the Breg is to regulate immune responses and maintain tolerance (73). They achieve this primarily through the production of immunosuppressive cytokines like IL-10, as well as through other mechanisms.

The most important function of Breg is the suppression of T cell responses (84, 89). Breg play a crucial role in controlling T cell activity, especially CD4^+^ T helper (Th) cells (90–92). By producing IL-10, they inhibit the activation, proliferation, and cytokine production of effector T cells (Teff), thus preventing excessive immune responses and alleviating tissue damage (83, 85). They can also induce the differentiation of regulatory T cells (Tregs), which further enhances immune suppression (83, 91, 93). Meanwhile, Breg could regulate the inflammatory cytokines by suppressing the production of pro-inflammatory cytokines, such as TNF-α (94, 95), IL-6 (96), IL-17 (97, 98), and IFNγ (99).

## 5 Mechanisms of Breg in organ transplantation

Breg contribute to immune tolerance in organ transplantation through a variety of mechanisms, many of which revolve around their capacity to suppress excessive immune responses, inhibit T-cell activation, and promote an anti-inflammatory microenvironment (8, 100, 101). The main mechanisms are as follows:

### 5.1 Bregs mediate immune tolerance via anti-inflammatory cytokines

The primary function of Breg in transplantation is the secretion of anti-inflammatory cytokines, particularly IL-10. IL-10 suppresses immune responses in several ways. It could inhibit T-cell activation (102, 103). IL-10 directly suppresses the activation, proliferation, and cytokine production of effector T cells (both CD4^+^ and CD8^+^ T cells), preventing them from recognizing and attacking the transplanted organ (104, 105). Besides, it could stimulate the differentiation and expansion of regulatory T cells (Tregs) by IL-10 (106). Tregs, in turn, regulate both T cell-mediated and antibody-mediated rejection (107). Further, it could inhibit the antigen-presenting cells (APCs) (108). IL-10 downregulates the activity of APCs such as dendritic cells, macrophages, and B cells, reducing their capacity to stimulate T cell responses.

### 5.2 Regulation of B cell responses to reduce graft rejection

Breg also exert their immunosuppressive effects on B cells by modulating the activation and function of other B cell subsets. In transplantation, the role of B cells can be complex, as they contribute to both humoral rejection (antibody-mediated rejection) and immune regulation (109). In transplantation, Breg help suppress the activation of autoreactive B cells that produce antibodies against the graft (110). By reducing the production of alloantibodies (antibodies that recognize donor antigens), Breg help prevent antibody-mediated rejection (111). Furthermore, it controls antigen-specific B cell responses. Breg can inhibit the expansion and differentiation of antigen-specific B cells that produce graft-specific antibodies, which could otherwise contribute to graft rejection. To induce allograft tolerance, Bregs can be induced by anti-CD45RB and anti-TIM1antibody, which means that Breg requires antigen recognition for tolerance inducition (112).

### 5.3 Direct suppression of T cell-mediated rejection

In transplantation, Breg can directly suppress T cell responses via cell-to-cell contact, in addition to cytokine secretion. The first way is to induce immune checkpoint proteins. Breg expresses inhibitory molecules like PD-L1 (113–115), CTLA-4 (84), and TIGIT (116), which can interact with their respective ligands on T cells to induce immune suppression. These interactions inhibit T-cell activation and promote tolerance. The second way is via the induction of anergy in T cells. Breg can induce anergy in CD4^+^ and CD8^+^ T cells through direct interactions, preventing them from responding to allo-antigens (117). The third way is via the induction of Treg differentiation. Through direct contact, Breg can promote the conversion of naïve T cells into regulatory T cells (Tregs), which are crucial for maintaining immune tolerance to the graft (91).

### 5.4 Induction of graft-specific tolerance

Breg is involved in the establishment and maintenance of graft-specific tolerance, which is essential for long-term organ survival without the need for chronic immunosuppression (2). Breg could induce donor-specific tolerance by promoting the tolerance specifically to donor antigens. This may involve the promotion of Tregs or other regulatory cells that target graft-specific immune responses, allowing the recipient’s immune system to accept the transplanted organ as “self” (2). Furthermore, Breg could regulate inflammatory responses in the transplant microenvironment (87, 118). Breg helps to create an anti-inflammatory environment within the graft, which reduces the activation of both innate and adaptive immune responses that could lead to graft rejection (7, 8, 101, 119).

### 5.5 Interactions between Bregs and other immune cells in transplantation

By interacting with other immune cells, Breg could stimulate tolerance in transplantation. Breg can modulate dendritic cell (DC) function, reducing their ability to activate T cells. By interacting with DCs, Breg can decrease the presentation of donor antigens and thereby lower the risk of graft rejection (83). Breg could mitigate the function of macrophages, which play a central role in transplant rejection and immune surveillance (120). By reducing macrophage activation, Breg can help prevent tissue damage in the graft. For the natural killer (NK) cells, there is emerging evidence suggesting that Breg may also interact with NK cells, which are involved in innate immunity and can contribute to graft rejection. Breg can inhibit NK cell cytotoxicity and promote immune tolerance in the transplantation (121).

## 6 Breg in solid organ transplantation

### 6.1 Renal transplantation

In solid organ transplantation, Breg contributes to the induction of tolerance and the prevention of both acute and chronic rejection (8, 122, 123). Studies in mouse models of renal transplantation show that Breg play a role in the tolerance of the grafts by modulating T cell responses and promoting IL-10 production (73, 124). In a cohort of 200 kidney transplant recipients, an imbalance of circulating follicular helper T cells (cTfh) over IL10^+^ Breg leads to graft failure. Meanwhile, the increase in the cTfh/IL10^+^Breg ratio is an index of acute rejection (125). A cohort of human renal transplantation with calcineurin inhibitors (CNI) or mammalian target of rapamycin (mTOR) inhibitors showed that CD19^+^CD24^hi^CD38^hi^ Breg increases over time and contributes to the long-term graft survival is not correlated with these drugs (126). In human allogenic renal transplantation, Breg could regulate the IL-10 and TNF-α expression ratios to alleviate renal injury after transplantation (95). Moreover, in kidney transplant recipients, the levels of CD19^+^CD25^+^ Breg are positively correlated with better graft function and longer and higher Treg levels (14). In another study of human kidney allografts, human leukocyte antigen G (HLA-G) stimulates IL-10-producing memory Breg (CD19^+^CD24^hi^CD27^+^IL-10^+^) to prolong graft survival by decreasing CD3^+^ T cell proliferation (127). Renal transplant recipients could benefit from the induction of long-lasting CD19^+^CD24^hi^CD38^hi^Breg (128). In renal transplant recipients, a higher level of CD19^+^CD25^+^ Breg is independently associated with improved graft function (14). In a cohort of 58 kidney transplant recipients, IL-10-producing Breg could lead to non-immunosuppressant for at least 1 year after transplantation (129). Further, T1 and T2 transitional B cells (CD38^+^CD24^+^) were also increased in tolerant recipients. The 42 healthy controls also had IL-10-producing Breg. But they found no difference in TGF-β secreting B cells (129). In another cohort study with 71 kidney transplant recipients and 19 healthy controls, T1 and T2 transitional B cells (CD38^+^CD24^+^) were also increased in tolerant recipients, who had higher percentages of B cells and less NK and T cells. In the analysis of the tolerant drug-free patients, there is a redistribution of Breg, which produces TGF-β instead of IL-10 (130). In a Phase III clinical study of belatacept on kidney transplant recipients, the frequency and absolute number of transitional B cells, including CD19^+^CD24^hi^CD38^hi^ Breg and CD19^+^IgD^hi^CD38^hi^CD27^-^, and naïve B cells were significantly higher (131). Granzyme B-producing B cells are a characteristic B cell subset, identified with CD307b^hi^, CD258^hi^CD72^hi^, and CD21^lo^PD-1^hi^ B cell subpopulations (34). This subtype of Breg serves a dual function in renal transplantation. They act as regulatory cells to maintain allo-specific tolerance and as effector cells to enhance CMV viral control (132).

### 6.2 Other solid organ transplantation

Breg in liver transplant models has been shown to promote long-term graft survival by suppressing immune responses and promoting donor-specific tolerance (101). This is particularly important in liver transplantation, as the liver is considered to be an immunologically privileged organ, and Breg may help maintain this privilege (133). Meanwhile, the proportion of CD19^+^CD24^hi^CD27^+^ Breg has been reported to predict the occurrence of acute allograft rejection in liver transplantation (134). With the application of Sirolimus, both Breg and Treg are expanded in liver transplant patients (135).

For heart and lung transplantation, similar to kidney and liver transplantation, Breg contributes to immune regulation and graft survival in heart and lung transplant models. In the heart transplantation mouse model, histone deacetylase (HDAC) inhibitor trichostatin A (TSA) could increase the frequency of IL-10 and TGF-β-producing CD19^+^CD5^+^CD1d^hi^ Breg cells and thereby induce immune tolerance (136). With the adoptive transfer of the transplanted Breg in heart-transplanted mice, this Breg could induce transplantation tolerance via the CD40-TRAF6 signaling pathway in DCs (137). In a study of 117 cases of clinical lung transplantation recipients, CD19^+^CD24^hi^CD38^hi^ B-cells contribute to the long-term lung grafts survival (20).

For allogenic islet transplantation, two subsets of Breg play a key role in tolerance induction and maintenance. CD19^+^CD5^+^CD1d^+^ B10 cells are mainly responsible for the early stage of transplantation tolerance induction, and CD19^+^TIM-1^+^B cells are critical in the whole process of tolerance induction and maintenance (16). Another study on islet transplant tolerance revealed that Breg-dependent tolerance is dependent on NK cells (121). In a mismatched islet transplantation model, to establish transplantation tolerance, adoptively transferred Breg cells need the presence of Treg (73).

## 7 Conclusion and future perspective

**Figure 1:**
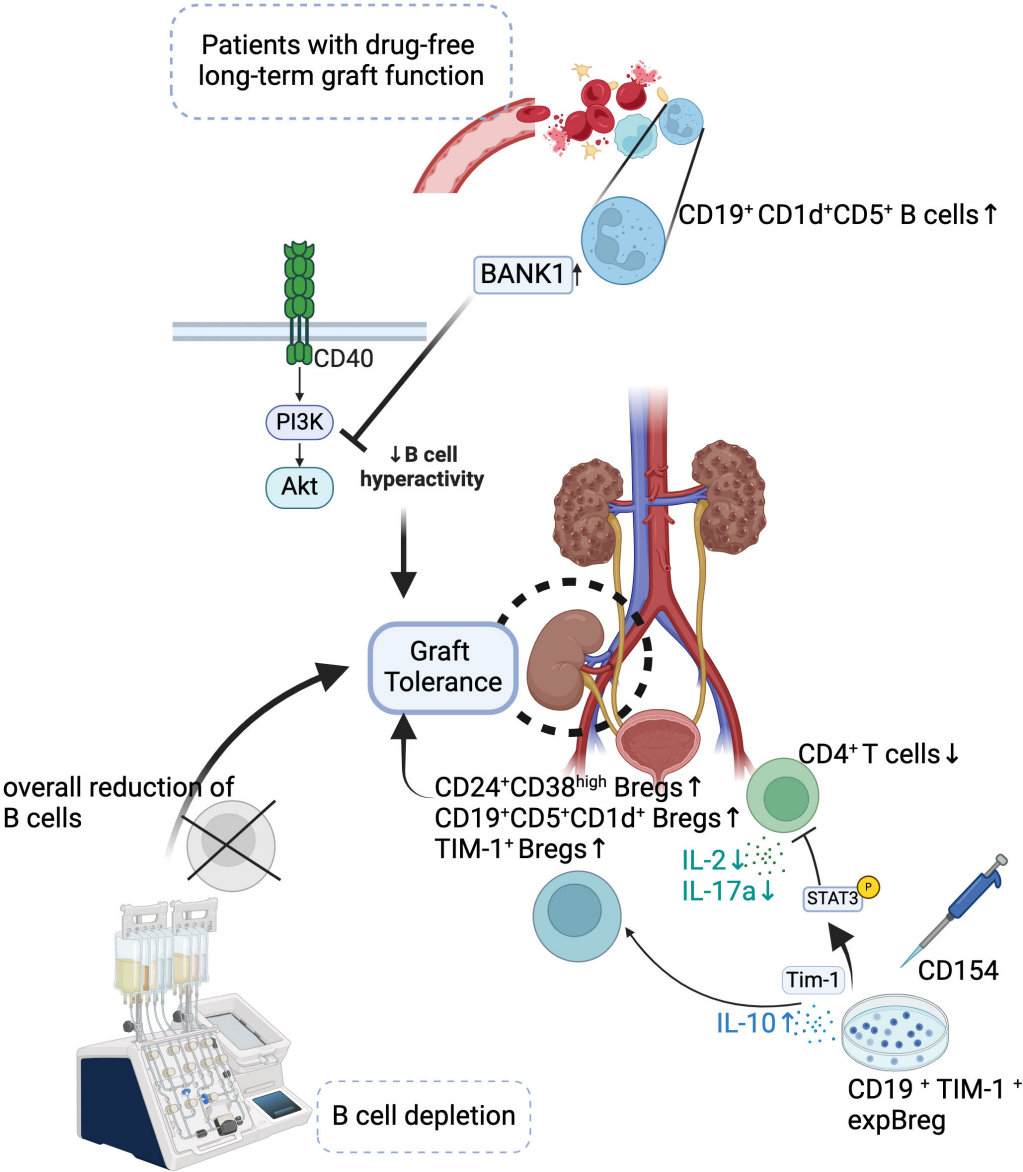
Mechanisms of Bregs in Promoting Graft Tolerance. Bregs contribute to long-term drug-free graft tolerance in transplant patients. Increased populations of CD24^+^CD38hi Bregs, CD19^+^CD1d^+^CD5^+^ B cells, and TIM-1^+^ Bregs, along with reduced CD4^+^ T cells, promote graft tolerance. BANK1-mediated inhibition of PI3K-Akt signaling reduces B cell hyperactivity, enhancing tolerance. Approaches like B cell depletion or expansion of TIM-1^+^ Bregs (expBregs) further support graft survival.

Breg in immune regulation as Tregs, especially in organ transplantation, offers significant therapeutic potential and provides promising potential for Breg-based therapies (138). Enhancing the function or expansion of Breg could be a promising therapeutic strategy to induce tolerance and promote graft survival (8, 139). It could be a substitute for immunosuppressive drugs, which may have significant adverse effects (140) (**Figure 1**).

Despite the promising role of Breg in organ transplantation, several challenges remain. One major unresolved issue is the heterogeneity of Breg subsets and the lack of standardized markers for their identification, making their clinical translation challenging. Additionally, while Breg-based therapies hold potential for inducing long-term tolerance, concerns remain regarding their stability, potential off-target effects, and the risk of over-suppressing the immune system. The optimal strategies for *in vivo* expansion or adoptive transfer of Breg also require further refinement. Future research should focus on defining the molecular mechanisms governing Breg differentiation and function, optimizing methods for their therapeutic application, and conducting long-term clinical studies to evaluate their efficacy in transplantation. Integrating Breg-based therapies with current immunosuppressive strategies may offer a novel approach to reducing drug toxicity while maintaining immune tolerance. Addressing these challenges will be crucial for advancing Breg-based immunotherapy in organ transplantation.

Overall, Breg plays a critical role in maintaining immune tolerance and promoting graft survival in organ transplantation. The therapeutic potential of Breg provides new hope for cell therapy in organ transplantation.

## References

[1] Black CK Termanini KM Aguirre O Hawksworth JS Sosin M. Solid organ transplantation in the 21(st) century. Ann Transl Med. (2018) 6:409. doi: 10.21037/atm.2018.09.68 30498736 PMC6230860

[2] Lee KM Fu Q Huai G Deng K Lei J Kojima L. Suppression of allograft rejection by regulatory B cells induced via TLR signaling. JCI Insight. (2022) 7:e152213. doi: 10.1172/jci.insight.152213 35943811 PMC9536278

[3] Pallardo Mateu LM Sancho Calabuig A Capdevila Plaza L Franco Esteve A. Acute rejection and late renal transplant failure: risk factors and prognosis. Nephrol Dial Transplant. (2004) 19 Suppl 3:iii38–42. doi: 10.1093/ndt/gfh1013 15192134

[4] Choudhary NS Saigal S Bansal RK Saraf N Gautam D Soin AS. Acute and chronic rejection after liver transplantation: what A clinician needs to know. J Clin Exp Hepatol. (2017) 7:358–66. doi: 10.1016/j.jceh.2017.10.003 PMC5715482 29234201

[5] Takai S Tateno M Hirano T Kondo N Hirose S Yoshiki T. Increased IgE level as a marker of host-versus-graft disease: inhibition of this HVGD with a monoclonal antibody to IL-4. Cell Immunol. (1993) 149:1–10. doi: 10.1006/cimm.1993.1131 8513506

[6] Pallua S Giesinger J Oberguggenberger A Kemmler G Nachbaur D Clausen J. Impact of GvHD on quality of life in long-term survivors of haematopoietic transplantation. Bone Marrow Transplant. (2010) 45:1534–9. doi: 10.1038/bmt.2010.5 20228854

[7] van de Veen W Stanic B Wirz OF Jansen K Globinska A Akdis M. Role of regulatory B cells in immune tolerance to allergens and beyond. J Allergy Clin Immunol. (2016) 138:654–65. doi: 10.1016/j.jaci.2016.07.006 27596706

[8] McNee A Kannan A Jull P Shankar S. Expanding human breg for cellular therapy in transplantation: time for translation. Transplantation. (2024). doi: 10.1097/TP.0000000000005243 PMC12091222 39439021

[9] Dasgupta S Dasgupta S Bandyopadhyay M. Regulatory B cells in infection, inflammation, and autoimmunity. Cell Immunol. (2020) 352:104076. doi: 10.1016/j.cellimm.2020.104076 32143836

[10] Su QY Jiang ZQ Song XY Zhang SX. Regulatory B cells in autoimmune diseases: Insights and therapeutic potential. J Autoimmun. (2024) 149:103326. doi: 10.1016/j.jaut.2024.103326 39520834

[11] Achour A Simon Q Mohr A Seite JF Youinou P Bendaoud B. Human regulatory B cells control the T(FH) cell response. J Allergy Clin Immunol. (2017) 140:215–22. doi: 10.1016/j.jaci.2016.09.042 27865860

[12] Mohd Jaya FN Garcia SG Borras FE Chan GCF Franquesa M. Paradoxical role of Breg-inducing cytokines in autoimmune diseases. J Transl Autoimmun. (2019) 2:100011. doi: 10.1016/j.jtauto.2019.100011 32743499 PMC7388338

[13] Rosser EC Mauri C. Regulatory B cells: origin, phenotype, and function. Immunity. (2015) 42:607–12. doi: 10.1016/j.immuni.2015.04.005 25902480

[14] Ibrahim EH Aly MG Opelz G Morath C Zeier M Susal C. Higher CD19+CD25(+) Bregs are independently associated with better graft function in renal transplant recipients. BMC Nephrol. (2021) 22:180. doi: 10.1186/s12882-021-02374-2 33993874 PMC8127305

[15] Zhang M Zheng X Zhang J Zhu Y Zhu X Liu H. CD19(+)CD1d(+)CD5(+) B cell frequencies are increased in patients with tuberculosis and suppress Th17 responses. Cell Immunol. (2012) 274:89–97. doi: 10.1016/j.cellimm.2012.01.007 22361174

[16] Li S Li X Yang M Wei L Wei L Deng S. Identification of the subsets of IL-10-producing regulatory B cells in the course of tolerance induction and maintenance in islet allotransplantation. Transplant Proc. (2018) 50:3900–5. doi: 10.1016/j.transproceed.2018.04.065 30577284

[17] Simon Q Pers JO Cornec D Le Pottier L Mageed RA Hillion S. In-depth characterization of CD24(high)CD38(high) transitional human B cells reveals different regulatory profiles. J Allergy Clin Immunol. (2016) 137:1577–84 e10. doi: 10.1016/j.jaci.2015.09.014 26525227

[18] Sumimoto K Uchida K Kusuda T Mitsuyama T Sakaguchi Y Fukui T. The role of CD19+ CD24high CD38high and CD19+ CD24high CD27+ regulatory B cells in patients with type 1 autoimmune pancreatitis. Pancreatology. (2014) 14:193–200. doi: 10.1016/j.pan.2014.02.004 24854615

[19] Hayashi T Nakamae H Takeda S Nakashima Y Koh H Nishimoto M. Increasing numbers of CD19 + CD24(high)CD38(high) regulatory B cells and pre-germinal center B cells reflect activated autoimmunity and predict future treatment response in patients with untreated immune thrombocytopenia. Int J Hematol. (2021) 114:580–90. doi: 10.1007/s12185-021-03192-w 34309815

[20] Piloni D Morosini M Magni S Balderacchi A Inghilleri S Cova E. Peripheral CD19+CD24(high)CD38(high) B-regulatory cells in lung transplant recipients. Transpl Immunol. (2019) 57:101245. doi: 10.1016/j.trim.2019.101245 31526864

[21] Saraiva M O’Garra A. The regulation of IL-10 production by immune cells. Nat Rev Immunol. (2010) 10:170–81. doi: 10.1038/nri2711 20154735

[22] Lin X Wang X Xiao F Ma K Liu L Wang X. IL-10-producing regulatory B cells restrain the T follicular helper cell response in primary Sjogren’s syndrome. Cell Mol Immunol. (2019) 16:921–31. doi: 10.1038/s41423-019-0227-z PMC6884445 30948793

[23] Figueiro F Muller L Funk S Jackson EK Battastini AM Whiteside TL. Phenotypic and functional characteristics of CD39(high) human regulatory B cells (Breg). Oncoimmunology. (2016) 5:e1082703. doi: 10.1080/2162402X.2015.1082703 27057473 PMC4801473

[24] Sakkas LI Mavropoulos A Zafiriou E Roussaki-Schulze A Bogdanos DP. The effect of Apremilast on signal transduction and IL-10 production in CD39high regulatory B cells in patients with psoriatic arthritis. Mediterr J Rheumatol. (2018) 29:59–61. doi: 10.31138/mjr.29.1.59 32185301 PMC7045954

[25] Pati S Mukherjee S Dutta S Guin A Roy D Bose S. Tumor-associated CD19+CD39- B regulatory cells deregulate class-switch recombination to suppress antibody responses. Cancer Immunol Res. (2023) 11:364–80. doi: 10.1158/2326-6066.CIR-21-1073 36574614

[26] Wang RX Yu CR Dambuza IM Mahdi RM Dolinska MB Sergeev YV. Interleukin-35 induces regulatory B cells that suppress autoimmune disease. Nat Med. (2014) 20:633–41. doi: 10.1038/nm.3554 PMC4048323 24743305

[27] Choi JK Egwuagu CE. Interleukin 35 regulatory B cells. J Mol Biol. (2021) 433:166607. doi: 10.1016/j.jmb.2020.07.019 32755620 PMC7779660

[28] Luo Z Lundin S Mejia-Cordova M Hassani I Blixt M Hjelmqvist D. Interleukin-35 prevents development of autoimmune diabetes possibly by maintaining the phenotype of regulatory B cells. Int J Mol Sci. (2021) 22:12988. doi: 10.3390/ijms222312988 34884797 PMC8657454

[29] Ye Z Jiang Y Sun D Zhong W Zhao L Jiang Z. The plasma interleukin (IL)-35 level and frequency of circulating IL-35(+) regulatory B cells are decreased in a cohort of chinese patients with new-onset systemic lupus erythematosus. Sci Rep. (2019) 9:13210. doi: 10.1038/s41598-019-49748-z 31519970 PMC6744462

[30] Zhang Y Wei S Wu Q Shen X Dai W Zhang Z. Interleukin-35 promotes Breg expansion and interleukin-10 production in CD19(+) B cells in patients with ankylosing spondylitis. Clin Rheumatol. (2022) 41:2403–16. doi: 10.1007/s10067-022-06137-8 PMC9287221 35420296

[31] Li Q Yang C Liu C Zhang Y An N Ma X. The circulating IL-35(+) regulatory B cells are associated with thyroid associated opthalmopathy. Immun Inflammation Dis. (2024) 12:e1304. doi: 10.1002/iid3.v12.5 PMC11131934 38804861

[32] Egwuagu CE Yu CR. Interleukin 35-producing B cells (i35-breg): A new mediator of regulatory B-cell functions in CNS autoimmune diseases. Crit Rev Immunol. (2015) 35:49–57. doi: 10.1615/CritRevImmunol.2015012558 25746047 PMC5433835

[33] Liu Y Luo Y Zhu T Jiang M Tian Z Tang G. Regulatory B cells dysregulated T cell function in an IL-35-dependent way in patients with chronic hepatitis B. Front Immunol. (2021) 12:653198. doi: 10.3389/fimmu.2021.653198 33912178 PMC8072152

[34] Chesneau M Mai HL Danger R Le Bot S Nguyen TV Bernard J. Efficient expansion of human granzyme B-expressing B cells with potent regulatory properties. J Immunol. (2020) 205:2391–401. doi: 10.4049/jimmunol.2000335 32948686

[35] Floudas A Amu S Fallon PG. New insights into IL-10 dependent and IL-10 independent mechanisms of regulatory B cell immune suppression. J Clin Immunol. (2016) 36 Suppl 1:25–33. doi: 10.1007/s10875-016-0263-8 27008462

[36] Boonpiyathad T Satitsuksanoa P Akdis M Akdis CA. Il-10 producing T and B cells in allergy. Semin Immunol. (2019) 44:101326. doi: 10.1016/j.smim.2019.101326 31711770

[37] Nouel A Pochard P Simon Q Segalen I Le Meur Y Pers JO. B-Cells induce regulatory T cells through TGF-beta/IDO production in A CTLA-4 dependent manner. J Autoimmun. (2015) 59:53–60. doi: 10.1016/j.jaut.2015.02.004 25753821

[38] Xu L Liu X Liu H Zhu L Zhu H Zhang J. Impairment of granzyme B-producing regulatory B cells correlates with exacerbated rheumatoid arthritis. Front Immunol. (2017) 8:768. doi: 10.3389/fimmu.2017.00768 28713386 PMC5491972

[39] Chesneau M Le Mai H Brouard S. New method for the expansion of highly purified human regulatory granzyme B-expressing B cells. Methods Mol Biol. (2021) 2270:203–16. doi: 10.1007/978-1-0716-1237-8_11 33479900

[40] Bedke T Muscate F Soukou S Gagliani N Huber S. Title: IL-10-producing T cells and their dual functions. Semin Immunol. (2019) 44:101335. doi: 10.1016/j.smim.2019.101335 31734129

[41] Bhattacharyya S Sen P Wallet M Long B Baldwin AS Jr. Tisch R. Immunoregulation of dendritic cells by IL-10 is mediated through suppression of the PI3K/Akt pathway and of IkappaB kinase activity. Blood. (2004) 104:1100–9. doi: 10.1182/blood-2003-12-4302 15113757

[42] Ip WKE Hoshi N Shouval DS Snapper S Medzhitov R. Anti-inflammatory effect of IL-10 mediated by metabolic reprogramming of macrophages. Science. (2017) 356:513–9. doi: 10.1126/science.aal3535 PMC6260791 28473584

[43] Chernoff AE Granowitz EV Shapiro L Vannier E Lonnemann G Angel JB. A randomized, controlled trial of IL-10 in humans. Inhibition of inflammatory cytokine production and immune responses. J Immunol. (1995) 154:5492–9. doi: 10.4049/jimmunol.154.10.5492 7730651

[44] Ouyang W Rutz S Crellin NK Valdez PA Hymowitz SG. Regulation and functions of the IL-10 family of cytokines in inflammation and disease. Annu Rev Immunol. (2011) 29:71–109. doi: 10.1146/annurev-immunol-031210-101312 21166540

[45] Cope A Le Friec G Cardone J Kemper C. The Th1 life cycle: molecular control of IFN-gamma to IL-10 switching. Trends Immunol. (2011) 32:278–86. doi: 10.1016/j.it.2011.03.010 21531623

[46] Fiorentino DF Zlotnik A Vieira P Mosmann TR Howard M Moore KW. IL-10 acts on the antigen-presenting cell to inhibit cytokine production by Th1 cells. J Immunol. (1991) 146:3444–51. doi: 10.4049/jimmunol.146.10.3444 1827484

[47] Steensberg A Fischer CP Keller C Moller K Pedersen BK. IL-6 enhances plasma IL-1ra, IL-10, and cortisol in humans. Am J Physiol Endocrinol Metab. (2003) 285:E433–7. doi: 10.1152/ajpendo.00074.2003 12857678

[48] Kuwata H Watanabe Y Miyoshi H Yamamoto M Kaisho T Takeda K. IL-10-inducible Bcl-3 negatively regulates LPS-induced TNF-alpha production in macrophages. Blood. (2003) 102:4123–9. doi: 10.1182/blood-2003-04-1228 12907458

[49] Royer B Varadaradjalou S Saas P Guillosson JJ Kantelip JP Arock M. Inhibition of IgE-induced activation of human mast cells by IL-10. Clin Exp Allergy. (2001) 31:694–704. doi: 10.1046/j.1365-2222.2001.01069.x 11422128

[50] Kennedy Norton S Barnstein B Brenzovich J Bailey DP Kashyap M Speiran K. IL-10 suppresses mast cell IgE receptor expression and signaling *in vitro* and *in vivo* . J Immunol. (2008) 180:2848–54. doi: 10.4049/jimmunol.180.5.2848 18292506

[51] Wu K Bi Y Sun K Wang C. IL-10-producing type 1 regulatory T cells and allergy. Cell Mol Immunol. (2007) 4:269–75. 17764617

[52] Itoh K Hirohata S. The role of IL-10 in human B cell activation, proliferation, and differentiation. J Immunol. (1995) 154:4341–50. doi: 10.4049/jimmunol.154.9.4341 7722292

[53] Dambuza IM He C Choi JK Yu CR Wang R Mattapallil MJ. IL-12p35 induces expansion of IL-10 and IL-35-expressing regulatory B cells and ameliorates autoimmune disease. Nat Commun. (2017) 8:719. doi: 10.1038/s41467-017-00838-4 28959012 PMC5620058

[54] Yu CR Choi JK Uche AN Egwuagu CE. Production of IL-35 by Bregs is mediated through binding of BATF-IRF-4-IRF-8 complex to il12a and ebi3 promoter elements. J Leukoc Biol. (2018) 104:1147–57. doi: 10.1002/JLB.3A0218-071RRR PMC11290588 30117603

[55] Saheb-Sharif-Askari F Zakri AM Alenazy MF El-Wetidy MS Khalid-Salah-Al-Sheakly B Saheb-Sharif-Askari N. IL-35 promotes IL-35(+)IL-10(+) Bregs and Conventional LAG3(+) Tregs in the lung tissue of OVA-Induced Asthmatic Mice. Inflammation Res. (2024) 73:1699–709. doi: 10.1007/s00011-024-01925-1 39127869

[56] Xu WL Wang RL Liu Z Wu Q Li XL He Q. Granzyme B-producing B cells function as a feedback loop for T helper cells in liver transplant recipients with acute rejection. Inflammation. (2021) 44:2270–8. doi: 10.1007/s10753-021-01498-9 34120305

[57] Sailliet N Mai HL Dupuy A Tilly G Fourgeux C Braud M. Human granzyme B regulatory B cells prevent effector CD4+CD25- T cell proliferation through a mechanism dependent from lymphotoxin alpha. Front Immunol. (2023) 14:1183714. doi: 10.3389/fimmu.2023.1183714 37588598 PMC10425555

[58] Ahsan NF Lourenco S Psyllou D Long A Shankar S Bashford-Rogers R. The current understanding of the phenotypic and functional properties of human regulatory B cells (Bregs). Oxf Open Immunol. (2024) 5:iqae012. doi: 10.1093/oxfimm/iqae012 39346706 PMC11427547

[59] Rickert RC. New insights into pre-BCR and BCR signalling with relevance to B cell Malignancies. Nat Rev Immunol. (2013) 13:578–91. doi: 10.1038/nri3487 23883968

[60] Ying H Li Z Yang L Zhang J. Syk mediates BCR- and CD40-signaling integration during B cell activation. Immunobiology. (2011) 216:566–70. doi: 10.1016/j.imbio.2010.09.016 PMC3075491 21074890

[61] Fowler N Davis E. Targeting B-cell receptor signaling: changing the paradigm. Hematol Am Soc Hematol Educ Program. (2013) 2013:553–60. doi: 10.1182/asheducation-2013.1.553 24319231

[62] Naughton R Quiney C Turner SD Cotter TG. Bcr-Abl-mediated redox regulation of the PI3K/AKT pathway. Leukemia. (2009) 23:1432–40. doi: 10.1038/leu.2009.49 19295548

[63] Gallego-Valle J Perez-Fernandez VA Correa-Rocha R Pion M. Generation of human breg-like phenotype with regulatory function *in vitro* with bacteria-derived oligodeoxynucleotides. Int J Mol Sci. (2018) 19. doi: 10.3390/ijms19061737 PMC6032322 29895745

[64] Bishop GA Hostager BS. Signaling by CD40 and its mimics in B cell activation. Immunol Res. (2001) 24:97–109. doi: 10.1385/IR:24:2:097 11594459

[65] Weller S Faili A Garcia C Braun MC Le Deist FF de Saint Basile GG. CD40-CD40L independent Ig gene hypermutation suggests a second B cell diversification pathway in humans. Proc Natl Acad Sci U S A. (2001) 98:1166–70. doi: 10.1073/pnas.98.3.1166 PMC14726 11158612

[66] Zarnegar B He JQ Oganesyan G Hoffmann A Baltimore D Cheng G. Unique CD40-mediated biological program in B cell activation requires both type 1 and type 2 NF-kappaB activation pathways. Proc Natl Acad Sci U S A. (2004) 101:8108–13. doi: 10.1073/pnas.0402629101 PMC419565 15148378

[67] Chen D Ireland SJ Remington G Alvarez E Racke MK Greenberg B. CD40-mediated NF-kappaB activation in B cells is increased in multiple sclerosis and modulated by therapeutics. J Immunol. (2016) 197:4257–65. doi: 10.4049/jimmunol.1600782 PMC5312703 27798157

[68] Iwata S Hajime Sumikawa M Tanaka Y. B cell activation via immunometabolism in systemic lupus erythematosus. Front Immunol. (2023) 14:1155421. doi: 10.3389/fimmu.2023.1155421 37256149 PMC10225689

[69] Lee MB Lee JH Hong SH You JS Nam ST Kim HW. JQ1, a BET inhibitor, controls TLR4-induced IL-10 production in regulatory B cells by BRD4-NF-kappaB axis. BMB Rep. (2017) 50:640–6. doi: 10.5483/BMBRep.2017.50.12.194 PMC5749911 29187284

[70] Liu M Wei F Wang J Yu W Shen M Liu T. Myeloid-derived suppressor cells regulate the immunosuppressive functions of PD-1(-)PD-L1(+) Bregs through PD-L1/PI3K/AKT/NF-kappaB axis in breast cancer. Cell Death Dis. (2021) 12:465. doi: 10.1038/s41419-021-03745-1 33967272 PMC8107179

[71] Michee-Cospolite M Boudigou M Grasseau A Simon Q Mignen O Pers JO. Molecular mechanisms driving IL-10- producing B cells functions: STAT3 and c-MAF as underestimated central key regulators? Front Immunol. (2022) 13:818814. doi: 10.3389/fimmu.2022.818814 35359922 PMC8961445

[72] Noh J Noh G Kim HS Kim AR Choi WS. Allergen-specific responses of CD19(+)CD5(+)Foxp3(+) regulatory B cells (Bregs) and CD4(+)Foxp3(+) regulatory T cell (Tregs) in immune tolerance of cow milk allergy of late eczematous reactions. Cell Immunol. (2012) 274:109–14. doi: 10.1016/j.cellimm.2012.01.005 22398308

[73] Lee KM Stott RT Zhao G SooHoo J Xiong W Lian MM. TGF-beta-producing regulatory B cells induce regulatory T cells and promote transplantation tolerance. Eur J Immunol. (2014) 44:1728–36. doi: 10.1002/eji.201344062 PMC4048633 24700192

[74] Huai G Markmann JF Deng S Rickert CG. TGF-beta-secreting regulatory B cells: unsung players in immune regulation. Clin Transl Immunol. (2021) 10:e1270. doi: 10.1002/cti2.1270 PMC8017464 33815797

[75] Jiang J Qin T Zhang L Liu Q Wu J Dai R. IL-21 rescues the defect of IL-10-producing regulatory B cells and improves allergic asthma in DOCK8 deficient mice. Front Immunol. (2021) 12:695596. doi: 10.3389/fimmu.2021.695596 34867940 PMC8636116

[76] Wu Y van Besouw NM Shi Y Hoogduijn MJ Wang L Baan CC. The biological effects of IL-21 signaling on B-cell-mediated responses in organ transplantation. Front Immunol. (2016) 7:319. doi: 10.3389/fimmu.2016.00319 27602031 PMC4994014

[77] Wang YH Tsai DY Ko YA Yang TT Lin IY Hung KH. Blimp-1 contributes to the development and function of regulatory B cells. Front Immunol. (2019) 10:1909. doi: 10.3389/fimmu.2019.01909 31474988 PMC6702260

[78] van der Vlugt LE Haeberlein S de Graaf W Martha TE Smits HH. Toll-like receptor ligation for the induction of regulatory B cells. Methods Mol Biol. (2014) 1190:127–41. doi: 10.1007/978-1-4939-1161-5_10 25015278

[79] Zhou M Wen Z Cheng F Ma J Li W Ren H. Tumor-released autophagosomes induce IL-10-producing B cells with suppressive activity on T lymphocytes via TLR2-MyD88-NF-kappaB signal pathway. Oncoimmunology. (2016) 5:e1180485. doi: 10.1080/2162402X.2016.1180485 27622036 PMC5006924

[80] Jahrsdorfer B Blackwell SE Wooldridge JE Huang J Andreski MW Jacobus LS. B-chronic lymphocytic leukemia cells and other B cells can produce granzyme B and gain cytotoxic potential after interleukin-21-based activation. Blood. (2006) 108:2712–9. doi: 10.1182/blood-2006-03-014001 PMC1895576 16809616

[81] Hagn M Sontheimer K Dahlke K Brueggemann S Kaltenmeier C Beyer T. Human B cells differentiate into granzyme B-secreting cytotoxic B lymphocytes upon incomplete T-cell help. Immunol Cell Biol. (2012) 90:457–67. doi: 10.1038/icb.2011.64 21808264

[82] Kaltenmeier C Gawanbacht A Beyer T Lindner S Trzaska T van der Merwe JA. CD4+ T cell-derived IL-21 and deprivation of CD40 signaling favor the *in vivo* development of granzyme B-expressing regulatory B cells in HIV patients. J Immunol. (2015) 194:3768–77. doi: 10.4049/jimmunol.1402568 25780036

[83] Mohib K Cherukuri A Zhou Y Ding Q Watkins SC Rothstein DM. Antigen-dependent interactions between regulatory B cells and T cells at the T:B border inhibit subsequent T cell interactions with DCs. Am J Transplant. (2020) 20:52–63. doi: 10.1111/ajt.15546 31355483 PMC8117747

[84] Kessel A Haj T Peri R Snir A Melamed D Sabo E. Human CD19(+)CD25(high) B regulatory cells suppress proliferation of CD4(+) T cells and enhance Foxp3 and CTLA-4 expression in T-regulatory cells. Autoimmun Rev. (2012) 11:670–7. doi: 10.1016/j.autrev.2011.11.018 22155204

[85] Gutierrez C Lopez-Abente J Perez-Fernandez V Prieto-Sanchez A Correa-Rocha R Moreno-Guillen S. Analysis of the dysregulation between regulatory B and T cells (Breg and Treg) in human immunodeficiency virus (HIV)-infected patients. PloS One. (2019) 14:e0213744. doi: 10.1371/journal.pone.0213744 30917149 PMC6436717

[86] Benedek G Zhang J Nguyen H Kent G Seifert H Vandenbark AA. Novel feedback loop between M2 macrophages/microglia and regulatory B cells in estrogen-protected EAE mice. J Neuroimmunol. (2017) 305:59–67. doi: 10.1016/j.jneuroim.2016.12.018 28284347 PMC5387865

[87] Flores-Borja F Blair P. Mechanisms of induction of regulatory B cells in the tumour microenvironment and their contribution to immunosuppression and pro-tumour responses. Clin Exp Immunol. (2022) 209:33–45. doi: 10.1093/cei/uxac029 35350071 PMC9307227

[88] Tousif S Wang Y Jackson J Hough KP Strenkowski JG Athar M. Indoleamine 2, 3-dioxygenase promotes aryl hydrocarbon receptor-dependent differentiation of regulatory B cells in lung cancer. Front Immunol. (2021) 12:747780. doi: 10.3389/fimmu.2021.747780 34867973 PMC8640488

[89] Rosser EC Blair PA Mauri C. Cellular targets of regulatory B cell-mediated suppression. Mol Immunol. (2014) 62:296–304. doi: 10.1016/j.molimm.2014.01.014 24556109

[90] Zhang Y Wu J Zhang H Wu C. The regulation between CD4(+)CXCR5(+) follicular helper T (Tfh) cells and CD19(+)CD24(hi)CD38(hi) regulatory B (Breg) cells in gastric cancer. J Immunol Res. (2022) 2022:9003902. doi: 10.1155/2022/9003902 36339942 PMC9629923

[91] Wang WW Yuan XL Chen H Xie GH Ma YH Zheng YX. CD19+CD24hiCD38hiBregs involved in downregulate helper T cells and upregulate regulatory T cells in gastric cancer. Oncotarget. (2015) 6:33486–99. doi: 10.18632/oncotarget.5588 PMC4741780 26378021

[92] Hong M Liao Y Liang J Chen X Li S Liu W. Immunomodulation of human CD19(+)CD25(high) regulatory B cells via Th17/Foxp3 regulatory T cells and Th1/Th2 cytokines. Hum Immunol. (2019) 80:863–70. doi: 10.1016/j.humimm.2019.05.011 31262519

[93] Luo Y Acevedo D Vlagea A Codina A Garcia-Garcia A Deya-Martinez A. Changes in Treg and Breg cells in a healthy pediatric population. Front Immunol. (2023) 14:1283981. doi: 10.3389/fimmu.2023.1283981 38077340 PMC10704817

[94] Poznansky SA Yu M Deng K Fu Q Markmann JF LeGuern C. Leveraging the tolerogenic potential of TNF-alpha and regulatory B cells in organ transplantation. Front Immunol. (2023) 14:1173672. doi: 10.3389/fimmu.2023.1173672 37180165 PMC10172648

[95] Cherukuri A Rothstein DM Clark B Carter CR Davison A Hernandez-Fuentes M. Immunologic human renal allograft injury associates with an altered IL-10/TNF-alpha expression ratio in regulatory B cells. J Am Soc Nephrol. (2014) 25:1575–85. doi: 10.1681/ASN.2013080837 PMC4073434 24610932

[96] Rosser EC Oleinika K Tonon S Doyle R Bosma A Carter NA. Regulatory B cells are induced by gut microbiota-driven interleukin-1beta and interleukin-6 production. Nat Med. (2014) 20:1334–9. doi: 10.1038/nm.3680 25326801

[97] Lee SY Lee SH Seo HB Ryu JG Jung K Choi JW. Inhibition of IL-17 ameliorates systemic lupus erythematosus in Roquin(san/san) mice through regulating the balance of TFH cells, GC B cells, Treg and Breg. Sci Rep. (2019) 9:5227. doi: 10.1038/s41598-019-41534-1 30914691 PMC6435653

[98] Mu Y Xu W Liu J Wang Y Chen J Zhou Q. Mesenchymal stem cells moderate experimental autoimmune uveitis by dynamic regulating Th17 and Breg cells response. J Tissue Eng Regener Med. (2022) 16:26–35. doi: 10.1002/term.v16.1 34674378

[99] Mavropoulos A Varna A Zafiriou E Liaskos C Alexiou I Roussaki-Schulze A. IL-10 producing Bregs are impaired in psoriatic arthritis and psoriasis and inversely correlate with IL-17- and IFNgamma-producing T cells. Clin Immunol. (2017) 184:33–41. doi: 10.1016/j.clim.2017.04.010 28461105

[100] Beckett J Hester J Issa F Shankar S. Regulatory B cells in transplantation: roadmaps to clinic. Transpl Int. (2020) 33:1353–68. doi: 10.1111/tri.v33.11 32725703

[101] Elias C Chen C Cherukuri A. Regulatory B cells in solid organ transplantation: from immune monitoring to immunotherapy. Transplantation. (2024) 108:1080–9. doi: 10.1097/TP.0000000000004798 PMC10985051 37779239

[102] Bouaziz JD Calbo S Maho-Vaillant M Saussine A Bagot M Bensussan A. IL-10 produced by activated human B cells regulates CD4(+) T-cell activation *in vitro* . Eur J Immunol. (2010) 40:2686–91. doi: 10.1002/eji.201040673 20809522

[103] Engelhardt KR Shah N Faizura-Yeop I Kocacik Uygun DF Frede N Muise AM. Clinical outcome in IL-10- and IL-10 receptor-deficient patients with or without hematopoietic stem cell transplantation. J Allergy Clin Immunol. (2013) 131:825–30. doi: 10.1016/j.jaci.2012.09.025 23158016

[104] Kingsley CI Karim M Bushell AR Wood KJ. CD25+CD4+ regulatory T cells prevent graft rejection: CTLA-4- and IL-10-dependent immunoregulation of alloresponses. J Immunol. (2002) 168:1080–6. doi: 10.4049/jimmunol.168.3.1080 11801641

[105] Blazar BR Taylor PA Panoskaltsis-Mortari A Narula SK Smith SR Roncarolo MG. Interleukin-10 dose-dependent regulation of CD4+ and CD8+ T cell-mediated graft-versus-host disease. Transplantation. (1998) 66:1220–9. doi: 10.1097/00007890-199811150-00018 9825821

[106] Kim YH Wee YM Choi MY Lim DG Kim SC Han DJ. Interleukin (IL)-10 induced by CD11b(+) cells and IL-10-activated regulatory T cells play a role in immune modulation of mesenchymal stem cells in rat islet allografts. Mol Med. (2011) 17:697–708. doi: 10.2119/molmed.2010.00098 21365122 PMC3146595

[107] Romano M Tung SL Smyth LA Lombardi G. Treg therapy in transplantation: a general overview. Transpl Int. (2017) 30:745–53. doi: 10.1111/tri.2017.30.issue-8 28012226

[108] Dai H Pena A Bauer L Williams A Watkins SC Camirand G. Treg suppression of immunity within inflamed allogeneic grafts. JCI Insight. (2022) 7:e160579. doi: 10.1172/jci.insight.160579 35881490 PMC9462475

[109] Wortel CM Heidt S. Regulatory B cells: Phenotype, function and role in transplantation. Transpl Immunol. (2017) 41:1–9. doi: 10.1016/j.trim.2017.02.004 28257995

[110] Berthelot JM Jamin C Amrouche K Le Goff B Maugars Y Youinou P. Regulatory B cells play a key role in immune system balance. Joint Bone Spine. (2013) 80:18–22. doi: 10.1016/j.jbspin.2012.04.010 22858147

[111] Zahran AM Elsayh KI Saad K Embaby M Ali AM. Regulatory B cells (CD19(+)CD38(hi)CD24(hi)) in alloimmunized and non-alloimmunized children with beta-thalassemia major. Blood Cells Mol Dis. (2016) 57:91–6. doi: 10.1016/j.bcmd.2016.01.005 26852663

[112] Kimura S Rickert CG Kojima L Aburawi M Tanimine N Fontan F. Regulatory B cells require antigen recognition for effective allograft tolerance induction. Am J Transplant. (2020) 20:977–87. doi: 10.1111/ajt.15739 PMC7372932 31823520

[113] Wu H Xia L Jia D Zou H Jin G Qian W. PD-L1(+) regulatory B cells act as a T cell suppressor in a PD-L1-dependent manner in melanoma patients with bone metastasis. Mol Immunol. (2020) 119:83–91. doi: 10.1016/j.molimm.2020.01.008 32001420

[114] Khan AR Hams E Floudas A Sparwasser T Weaver CT Fallon PG. PD-L1hi B cells are critical regulators of humoral immunity. Nat Commun. (2015) 6:5997. doi: 10.1038/ncomms6997 25609381

[115] Wang X Wang G Wang Z Liu B Han N Li J. PD-1-expressing B cells suppress CD4(+) and CD8(+) T cells via PD-1/PD-L1-dependent pathway. Mol Immunol. (2019) 109:20–6. doi: 10.1016/j.molimm.2019.02.009 30851633

[116] Hasan MM Nair SS O’Leary JG Thompson-Snipes L Nyarige V Wang J. Implication of TIGIT(+) human memory B cells in immune regulation. Nat Commun. (2021) 12:1534. doi: 10.1038/s41467-021-21413-y 33750787 PMC7943800

[117] Tretter T Venigalla RK Eckstein V Saffrich R Sertel S Ho AD. Induction of CD4+ T-cell anergy and apoptosis by activated human B cells. Blood. (2008) 112:4555–64. doi: 10.1182/blood-2008-02-140087 18802006

[118] Zhao D Huai G Yuan Y Cui Y Yuan Y Zhao G. Expansion of B10 cells *in vitro*: Pathways, techniques and applications in transplantation (Review). Int J Mol Med. (2025) 55(2):29. doi: 10.3892/ijmm.2024.5470 39670296 PMC11670864

[119] Peng B Ming Y Yang C. Regulatory B cells: the cutting edge of immune tolerance in For the generation of granzyme B-producing Breg. Cell Death Dis. (2018) 9:109. doi: 10.1038/s41419-017-0152-y 29371592 PMC5833552

[120] Chu Z Zou W Xu Y Sun Q Zhao Y. The regulatory roles of B cell subsets in transplantation. Expert Rev Clin Immunol. (2018) 14:115–25. doi: 10.1080/1744666X.2018.1426461 29338551

[121] Schuetz C Lee KM Scott R Kojima L Washburn L Liu L. Regulatory B cell-dependent islet transplant tolerance is also natural killer cell dependent. Am J Transplant. (2017) 17:1656–62. doi: 10.1111/ajt.14265 PMC5444975 28296255

[122] Long W Zhang H Yuan W Lan G Lin Z Peng L. The role of regulatory B cells in kidney diseases. Front Immunol. (2021) 12:683926. doi: 10.3389/fimmu.2021.683926 34108975 PMC8183681

[123] Oleinika K Mauri C Salama AD. Effector and regulatory B cells in immune-mediated kidney disease. Nat Rev Nephrol. (2019) 15:11–26. doi: 10.1038/s41581-018-0074-7 30443016

[124] Luo Y Luo F Zhang K Wang S Zhang H Yang X. Elevated circulating IL-10 producing breg, but not regulatory B cell levels, restrain antibody-mediated rejection after kidney transplantation. Front Immunol. (2020) 11:627496. doi: 10.3389/fimmu.2020.627496 33584730 PMC7877339

[125] Laguna-Goya R Utrero-Rico A Cano-Romero FL Gomez-Massa E Gonzalez E Andres A. Imbalance favoring follicular helper T cells over IL10(+) regulatory B cells is detrimental for the kidney allograft. Kidney Int. (2020) 98:732–43. doi: 10.1016/j.kint.2020.02.039 32495741

[126] Latorre I Esteve-Sole A Redondo D Giest S Argilaguet J Alvarez S. Calcineurin and mTOR inhibitors have opposing effects on regulatory T cells while reducing regulatory B cell populations in kidney transplant recipients. Transpl Immunol. (2016) 35:1–6. doi: 10.1016/j.trim.2016.01.004 26836476

[127] Ajith A Mamouni K Musa A Horuzsko DD Gani I Mulloy LL. IL-10-producing memory B regulatory cells as a novel target for HLA-G to prolong human kidney allograft survival. Hum Immunol. (2023) 84:366–73. doi: 10.1016/j.humimm.2023.03.003 36934068

[128] Morath C Schaier M Ibrahim E Wang L Kleist C Opelz G. Induction of long-lasting regulatory B lymphocytes by modified immune cells in kidney transplant recipients. J Am Soc Nephrol. (2023) 34:160–74. doi: 10.1681/ASN.2022020210 PMC10101591 36137752

[129] Newell KA Asare A Kirk AD Gisler TD Bourcier K Suthanthiran M. Identification of a B cell signature associated with renal transplant tolerance in humans. J Clin Invest. (2010) 120:1836–47. doi: 10.1172/JCI39933 PMC2877933 20501946

[130] Sagoo P Perucha E Sawitzki B Tomiuk S Stephens DA Miqueu P. Development of a cross-platform biomarker signature to detect renal transplant tolerance in humans. J Clin Invest. (2010) 120:1848–61. doi: 10.1172/JCI39922 PMC2877932 20501943

[131] Leibler C Matignon M Pilon C Montespan F Bigot J Lang P. Kidney transplant recipients treated with belatacept exhibit increased naive and transitional B cells. Am J Transplant. (2014) 14:1173–82. doi: 10.1111/ajt.12721 24730563

[132] Zhu J Zeng Y Dolff S Bienholz A Lindemann M Brinkhoff A. Granzyme B producing B-cells in renal transplant patients. Clin Immunol. (2017) 184:48–53. doi: 10.1016/j.clim.2017.04.016 28461110

[133] Zhou AW Jin J Liu Y. Cellular strategies to induce immune tolerance after liver transplantation: Clinical perspectives. World J Gastroenterol. (2024) 30:1791–800. doi: 10.3748/wjg.v30.i13.1791 PMC11036497 38659486

[134] Zhou H Zhan F Zhang H Gu J Mu X Gao J. The proportion of CD19(+)CD24(hi)CD27(+) regulatory B cells predicts the occurrence of acute allograft rejection in liver transplantation. Ann Transl Med. (2019) 7:465. doi: 10.21037/atm.2019.08.05 31700901 PMC6803179

[135] Song J Du G Chen W Bao P Li B Lu Q. The advantage of Sirolimus in amplifying regulatory B cells and regulatory T cells in liver transplant patients. Eur J Pharmacol. (2020) 869:172872. doi: 10.1016/j.ejphar.2019.172872 31846626

[136] Zhou B Mei F Wu C Xu H Liu Z Cui Y. Protective effect of trichostatin A on CD19(+)CD5(+)CD1d(high) regulatory B cells in heart transplantation. Mol Med Rep. (2021) 23(5):339. doi: 10.3892/mmr.2021.11978 33760120

[137] Li W Wang D Yue R Chen X Liu A Xu H. Gut microbes enlarged the protective effect of transplanted regulatory B cells on rejection of cardiac allografts. J Heart Lung Transplant. (2021) 40:1502–16. doi: 10.1016/j.healun.2021.08.008 34742645

[138] Bao Y Liu J Li Z Sun Y Chen J Ma Y. *Ex vivo*-generated human CD1c(+) regulatory B cells by a chemically defined system suppress immune responses and alleviate graft-versus-host disease. Mol Ther. (2024) 32:4372–82. doi: 10.1016/j.ymthe.2024.10.026 PMC11638867 39489917

[139] Mauri C Menon M. Human regulatory B cells in health and disease: therapeutic potential. J Clin Invest. (2017) 127:772–9. doi: 10.1172/JCI85113 PMC5330739 28248202

[140] de Mattos AM Olyaei AJ Bennett WM. Nephrotoxicity of immunosuppressive drugs: long-term consequences and challenges for the future. Am J Kidney Dis. (2000) 35:333–46. doi: 10.1016/S0272-6386(00)70348-9 10676738

